# Knowledge Mapping of the Links Between the Gut Microbiota and Heart Failure: A Scientometric Investigation (2006–2021)

**DOI:** 10.3389/fcvm.2022.882660

**Published:** 2022-04-28

**Authors:** Fei Mu, Meng Tang, Yue Guan, Rui Lin, Meina Zhao, Jiaxin Zhao, Shaojie Huang, Haiyue Zhang, Jingwen Wang, Haifeng Tang

**Affiliations:** ^1^Department of Pharmacy, Xijing Hospital, The Fourth Military Medical University, Xi’an, China; ^2^Department of Chinese Materia Medica and Natural Medicines, School of Pharmacy, The Fourth Military Medical University, Xi’an, China; ^3^Department of Pharmacy, Shaanxi University of Chinese Medicine, Xianyang, China; ^4^Department of Health Statistics, School of Preventive Medicine, The Fourth Military Medical University, Xi’an, China

**Keywords:** heart failure, gut microbiota, CiteSpace, VOS viewer, scientometric, frontier research hotspots

## Abstract

**Background:**

There is considerable research value and extensive application perspectives to explore the link between gut microbiota and heart failure. The purpose of this study is to provide an overview of overall characteristics, evolutionary pathways, frontier research hotspots, and future trends in this field.

**Methods:**

Research datasets were acquired from the Web of Science Core Collection (WoSCC) between January 1, 2006 and December 31, 2021. Three different analysis tools including one online platform, VOS viewer V1.6.17.0, and CiteSpace V5.8.R2 software were used in order to conduct collaboration network analysis, co-cited analysis, co-occurring analysis, and citation burst detection.

**Results:**

A total of 873 publications in the WoSCC database met the requirement. The overall characteristics analysis showed that a steady growth trend in the number of publications and citations, with the predominant literature type being articles and the most frequent subject category being cardiac cardiovascular systems. The United States was the most prolific country and the center of national collaboration. Cleveland Clinic and Nathalie M. Delzenne provided the leading influence with publications, the cooperation between the institutes and authors were relatively weak. Moreover, gut microbiota, heart failure, risk factor, obesity, and inflammation were the keywords that appeared more frequently in the clustering analysis of reference co-citation and keyword co-occurrence. Burst detection analysis of top keywords showed that trimethylamine *N*-oxide (TMAO), bile acid, blood pressure, hypertension, and fermentation were the new research foci on the association between gut microbiota and heart failure. Strategies to improve gut microbiota hold promise as a new approach to treat heart failure.

**Conclusion:**

The comprehensive bibliometric study indicates that the structured information may be helpful in understanding research trends in the link between gut microbiota and heart failure, and locating research hotspots and gaps in this domain, especially further advances in this field will lead to significant breakthroughs in the development of novel therapeutic tools for metabolic modulation of heart failure.

## Introduction

Heart failure, the terminal stage of many cardiovascular diseases, affects approximately 40 million people in the world (1). It is well known that people can suffer from heart failure for a variety of reasons, but the most common risk factors for heart failure are hypertension, coronary artery disease, obesity, diabetes, smoking, and genetics (1, 2). The “gut hypothesis” in heart failure has been prevalent for many years, arguing that the heart failure is exacerbated by the translocation of gut microbiota and elevated levels of circulating endotoxins caused by intestinal ischemia and congestion, suggesting an inevitable link between the gut microbiota and its metabolites and the pathogenesis of heart failure (3, 4). Of interest is the fact that research in this field is still in a phase of rapid exploratory development.

**FIGURE 1:**
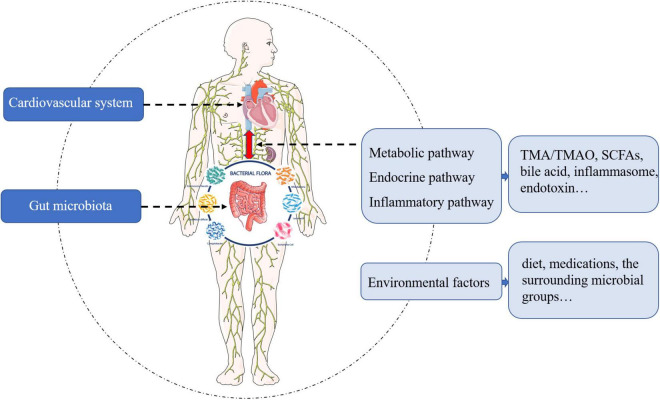
The pathways and mediators of bidirectional communication between cardiovascular system and gut microbiota.

Despite the fact that the bidirectional communication pathways between the gut microbiota and the cardiovascular system are not completely understood, there appears to be four main pathways (Figure 1). Impaired intestinal barrier function and inflammation levels, including high serum endotoxin, lipopolysaccharide, and cytokine levels, have been reported in patients with heart failure (5, 6). Gut microbiota and its metabolites, such as trimethylamine (TMA)/trimethylamine *N*-oxide (TMAO), short-chain fatty acids (SCFAs), and bile acids, also influence the host inflammation and cardiac biofunctions. In addition, studies have shown that environmental factors – diet, medications, and the surrounding milieu – play substantial roles in shaping our gut microbiome. Therefore, a deeper understanding of the gut microbiota and its metabolites would help to tease out the beneficial effects on heart failure in this complex multi-level network.

Knowledge mapping is the scientometric analysis method that provides a visual representation of scientific knowledge, it can be used to explore, analyze, and summarize the process and structure of knowledge development in both spatial and temporal dimensions. This method provides a diverse perspective that is not available in traditional literature reviews and systematic reviews, and has been applied in many fields domestically and internationally (7). Previous bibliometric studies have focused on the gut microbiota in obesity (8), or the microbiome-gut-brain axis (9), or gut microbiota in depression (10), but have not addressed the gut microbiota in heart failure. The purpose of this study is to analyze the overall characteristics, evolutionary pathways, frontier research hotspots, and future research development trends of the link between gut microbiota and heart failure using bibliometric methods, aiming to promote the diversification, deepening, and internationalization of research in this field.

## Materials and Methods

### Data Source

**FIGURE 2:**
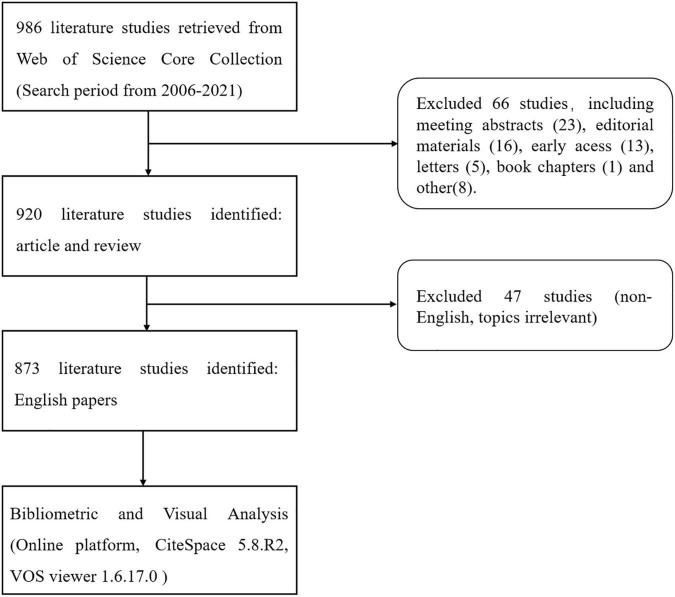
Flowchart of the inclusion and exclusion criteria.

The Web of Science Core Collection (WoSCC) is the most authoritative citation-based database with powerful indexing functions, and is widely used in scientometric analysis (11, 12). Therefore, this study selected to retrieve publications related to gut microbiota and heart failure in the WoSCC of Science Citation Index Expanded (SCIE). Our search terms combined medical subject headings words and keywords such as “heart failure” and “gut microbiota.” The full search terms are available in the study protocol (Supplementary Table 1). To avoid bias in data updates, the above operations were all executed within 1 day. A total of 986 results were found from January 1, 2006 to December 31, 2021 (retrieved on January 14, 2022). The type of literature studies was restricted to article or review, and literature language was set to English, with the specific inclusion and exclusion results shown in Figure 2. The Microsoft Excel 2019 and SPSS 23.0 were used to classify, descriptively analyze, and statistically evaluate the data extracted from the literatures. Moreover, the eligible literature studies were stored in download_txt format and exported for further use.

### Data Analysis and Visualization

Conceptual design of the study is presented in Supplementary Figure 1. All valid documents retrieved from WoSCC were converted to CiteSpace version 5.8.R2 (developed by Professor Chen Chaomei from Drexel University), VOS viewer version 1.6.17.0 (developed by Professor Eck and Waltman from Leiden University) and online platform^1^ for visual analysis (11, 13, 14). The parameters of CiteSpace were as follows: time span (2006–2021), years per slice (2), selection criteria (Top 50). In addition, VOS viewer software was used to conduct the network analysis of the frequent keywords. The parameters in the network analysis of the frequent keywords were as follows: the minimum number of occurrences of a keyword was 5 and resolution was 0.7. Descriptive indicators (number of publications and citations per year, literature types, subject categories, authors, and journals), relational indicators (collaborations of countries/regions, institutions, and authors), and qualitative indicators (bursts, betweenness centralities, and citation scores) were used in the assessment of bibliographic catalogs. We used a variety of scientometric methods, such as typical cluster analysis, co-citation analysis, and co-occurrence analysis, in order to identify intellectual structure, international collaborations, evolutionary pathways, as well as frontier research hotspots and future research development trends. In addition, some details of the setting description should be made known, where the ID #0 is assigned to the largest cluster formed in the cluster analysis, the ID #1 is assigned to the second largest, and so on.

## Results

### Analysis of Publication Outputs and Prediction of Growth Trend

**FIGURE 3:**
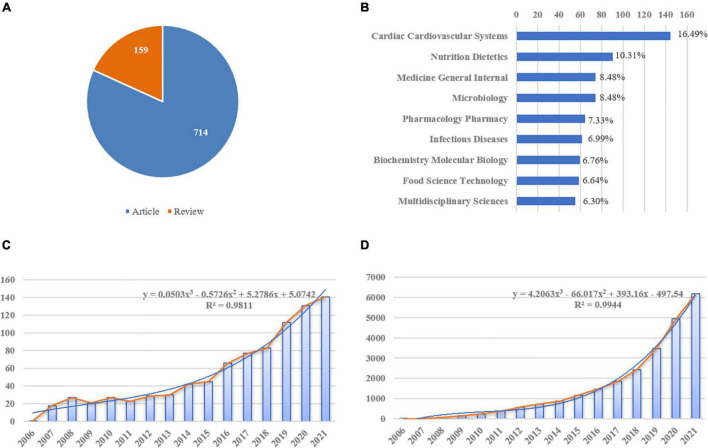
Yearly quantity and literature type of publications on gut microbiota and heart failure from 2006 to 2021. **(A)** Literature types distribution, the *blue* represents articles and *orange* represents reviews. **(B)** Subject categories distribution. **(C)** Annual publications quantitative distribution. **(D)** Annual cite distribution, the *blue* line represents the polynomial-fitted growth curve.

A total of 873 literature studies meeting the inclusion and exclusion criteria were finally retrieved for this study. A total of 714 (81.79%) articles and 159 (18.21%) reviews, respectively, were identified (Figure 3A). In addition, analysis of subject categories may provide insight into the subject focus of the current study, as shown in Figure 3B, there were top five subject categories in the analyzed publications: cardiac cardiovascular systems (*n* = 144, 16.49%), nutrition dietetics (*n* = 90, 10.31%), general internal medicine (*n* = 74, 8.48%), microbiology (*n* = 74, 8.48%), and pharmacology/pharmacy (*n* = 64, 7.33%). Over the past 16 years, the annual article productions had increased from 1 in 2006 to 141 in 2021, and also annual citations had steadily increased from 1 in 2006 to 6166 in 2021, indicating that related topic captures increasingly more attention from researchers. Furthermore, trend predictions were performed using linear, logarithmic, polynomial, power, exponential, and moving average function types. Figures 3C,D illustrated that the polynomial function provided the best fit to this prediction model as it has the highest *R*^2^ (0.9811, 0.9944, respectively), while the specific forecasting equations were *y* = 0.0503x^3^ − 0.5726x^2^ + 5.2786x + 5.0742 and *y* = 4.2063x^3^ − 66.017x^2^ + 393.16x − 497.54, respectively. Hence, ∼176 articles related to gut microbiota and heart failure may be published in 2022.

### Analysis of Scientific Collaboration Networks

**FIGURE 4:**
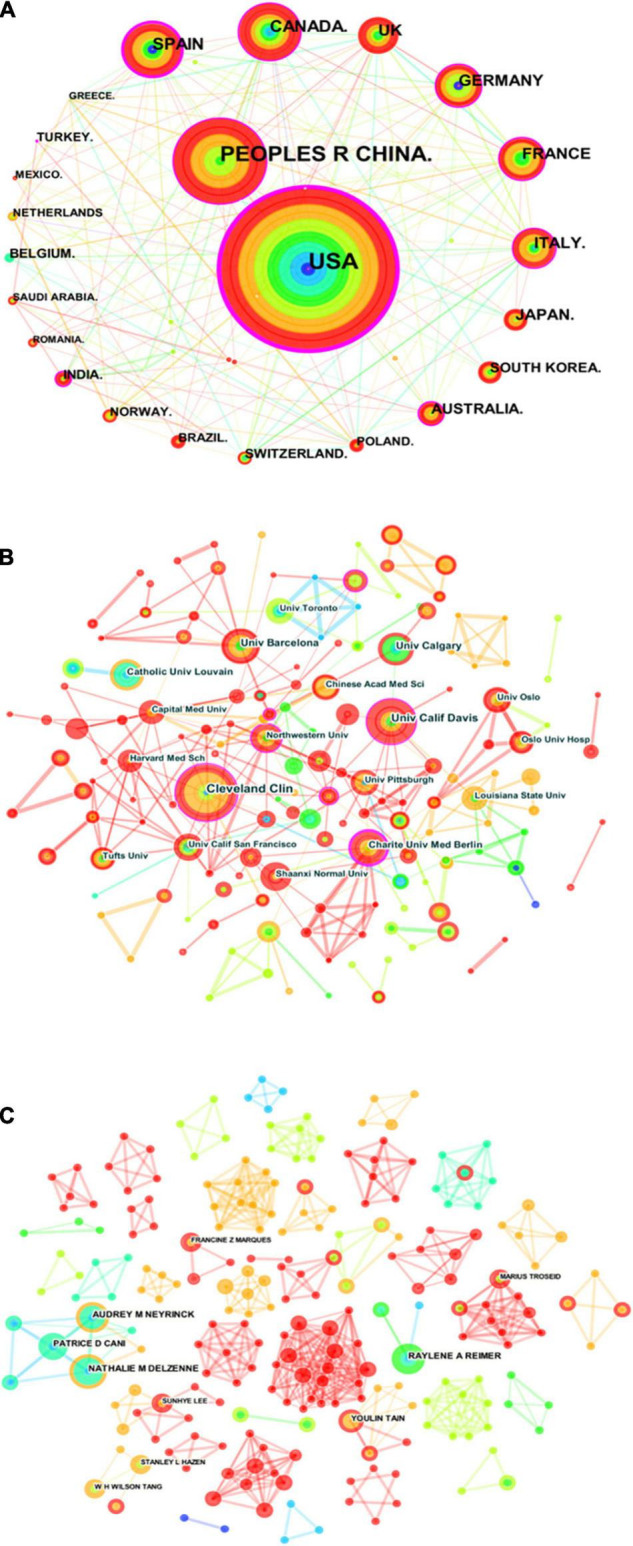
Visualization knowledge maps of the scientific collaboration network for gut microbiota research in the heart failure field between 2006 and 2021. **(A)** Inter-country collaboration network map. **(B)** Inter-institutional collaborative network map. **(C)** Inter-author collaborative network map. *Circle node* on the map represents a country, institute, or author, while *link lines* between nodes indicate collaborative relationships. The more published articles are, the larger their node area is. The outermost *purple ring* denotes the centrality level, and the nodes with high centrality are in the core position.

There were 40 countries, 196 institutions, and 257 authors involved in the publication on the relationship between gut microbiota and heart failure, respectively. Table 1 summarizes the top 10 high-yield countries, institutions and authors according to publications and centrality. The top five countries, in terms of the number of publications, were the United States, People’s Republic of China, Spain, Canada, and United Kingdom. In order of centrality, the United States, Spain, Canada, People’s Republic of China and India topped the list. The Pearson’s correlation analysis revealed that there was a significant correlation between publications and centrality at the country level (*r* = 0.859, *p* < 0.01). Figure 4A shows a map depicting the collaboration network among countries. Thus, taking a broader view of publications and centrality, the United States (publications: 281, centrality: 0.45) was identified as the most influential country in the field. Furthermore, the cross-country collaborations visualization map was generated by the online bibliometric analysis (Supplementary Figure 2), the results showed that the United States remains dominant. Figure 4B shows the cluster of institutions that performing gut microbiota research in heart failure. Its most productive institution was Cleveland Clinic with 16 publications, followed by University of California Davis (13), and University of Barcelona (11). Similarly, the Pearson’s correlation analysis concluded that there was a significant correlation between publications and centrality at the institutions level (*r* = 0.635, *p* < 0.01). In addition, three authors published more than eight publications in this field. The most productive author with nine publications was identified as Nathalie M. Delzenne, while Audrey M. Neyrinck (eight publications) and Raylene A. Reimer (eight publications) ranked second and third, respectively. There was a relative partial cooperation among Nathalie M. Delzenne, Audrey M. Neyrinck, and Patrice D. Cani (Figure 4C). However, the centrality between all of them was zero. Similarly, the VOS viewer software was also used to identify top countries, institutions and authors, and the results showed general agreement with the above results, as presented in Supplementary Figure 3.

**TABLE 1:** Ranking of the top 10 countries, institutions, and authors based on publications and centrality.

Items	Publications			Centrality		
	Ranking	Country	Number	Ranking	Name	Number
Country	1	United States	281	1	United States	0.45
	2	People’s Republic of China	167	2	Spain	0.21
	3	Spain	57	3	Canada	0.20
	4	Canada	56	4	People’s Republic of China	0.15
	5	United Kingdom	45	5	India	0.14
	6	France	44	6	Italy	0.13
	7	Germany	44	7	Australia	0.13
	8	Italy	42	8	France	0.12
	9	Japan	35	9	Germany	0.11
	10	Australia	32	10	Turkey	0.11
Institution	1	Cleveland Clinic	16	1	Charite-Medical University of Berlin	0.21
	2	University of California Davis	13	2	Cleveland Clinic	0.18
	3	University of Barcelona	11	3	Northwestern University	0.15
	4	University of Calgary	10	4	University of Washington	0.15
	5	Charite-Medical University of Berlin	9	5	University of British Columbia	0.15
	6	Catholic University of Louvain	9	6	University of California Davis	0.1
	7	Northwestern University	8	7	Capital Medical University	0.1
	8	University of California, San Francisco	8	8	McMaster University	0.1
	9	University of Toronto	8	9	University of Pittsburgh	0.09
	10	Chinese Academy of Medical Sciences	8	10	Harvard Medical School	0.09
Author	1	Nathalie M. Delzenne	9	1	Nathalie M. Delzenne	0
	2	Audrey M. Neyrinck	8	2	Audrey M. Neyrinck	0
	3	Raylene A. Reimer	8	3	Raylene A. Reimer	0
	4	Patrice D. Cani	7	4	Patrice D. Cani	0
	5	You-Lin Tain	6	5	You-Lin Tain	0
	6	Stanley L. Hazen	5	6	Stanley L. Hazen	0
	7	Marius Troseid	5	7	Marius Troseid	0
	8	Sunhye Lee	5	8	Sunhye Lee	0
	9	Francine Z. Marques	5	9	Francine Z. Marques	0
	10	W. H. Wilson Tang	5	10	W. H. Wilson Tang	0

### Analysis of Journals and Co-cited Journals

Journals are an important vehicle for presenting the results of academic research. Literature studies included in the study were published in 489 different journals, many of which were specialized journals. As shown in Table 2, the three most influential journals in terms of number of publications were *Plos One*, *Journal of Nutrition*, and *American Journal of Physiology-Gastrointestinal and Liver Physiology*. Most of the productive journals were in Q1, except for *European Journal of Clinical Microbiology and Infectious Disease*. As one of the most important metrics for scientometric investigation, co-citation analysis has been widely used. These co-cited journals were all in Q1 and had highly impact factor (IF), *PloS One* (408, IF = 3.240), *New England Journal of Medicine* (330, IF = 91.245), *Nature* (326, IF = 49.962), and *Circulation* (316, IF = 29.690) were top-ranked by citation counts. Meanwhile, visualization map of journal co-citation network was conducted to explore relationships between different journals from 2006 to 2021 (Supplementary Figure 4).

**TABLE 2:** Ranking of the top 10 journals and co-cited journals for gut microbiota research in the heart failure field.

Items	Ranking	Name	Country	Counts	IF (2020)^#^	JCR (2020)*
Journal	1	Plos One	United States	11	3.240	Q1
	2	Journal of Nutrition	United States	7	4.798	Q1
	3	American Journal of Physiology-Gastrointestinal and Liver Physiology	United States	6	4.052	Q1
	4	Food and Function	United Kingdom	6	5.396	Q1
	5	Journal of Nutritional Biochemistry	United States	6	6.048	Q1
	6	Molecular Nutrition and Food Research	Germany	6	5.914	Q1
	7	Nutrients	Switzerland	6	5.717	Q1
	8	Scientific Reports	United Kingdom	6	4.379	Q1
	9	British Journal of Nutrition	United Kingdom	5	3.718	Q1
	10	European Journal of Clinical Microbiology and Infectious Diseases	Germany	5	3.267	Q2
Co-cited Journal	1	Plos One	United States	408	3.240	Q1
	2	New England Journal of Medicine	United States	330	91.245	Q1
	3	Nature	United Kingdom	326	49.962	Q1
	4	Circulation	United States	316	29.690	Q1
	5	Proceedings of the National Academy of Sciences of the United States of America	United States	287	11.205	Q1
	6	Journal of the American College of Cardiology	Netherlands	278	24.094	Q1
	7	European Heart Journal	United Kingdom	228	29.983	Q1
	8	Lancet	United Kingdom	226	79.321	Q1
	9	Clinical Infectious Diseases	United Kingdom	214	9.079	Q1
	10	Science	United States	205	47.728	Q1

^#^IF, impact factor. *JCR, journal citation reports. Q, quartile in category.

### Analysis of Co-cited Authors

Co-cited author analysis can be used to identify research groups at “unknown” institutions or universities and to guide subsequent collaborations among scholars in the field. Supplementary Figure 5 is the co-citation author map, showing the collaboration between authors. As shown in Table 3, the top 10 co-cited authors, their affiliations, and their *h*-indexes were listed. In regard to the number of citations, Patrice D. Cani ranked first, with 145 citations, followed by W. H. Wilson Tang, Peter J. Turnbaugh, and Ruth E. Ley, whereas the remaining authors had fewer than 100. In terms of *h*-indexes, Patrice D. Cani also ranked first, followed by Fredrik Backhed and W. H. Wilson Tang.

**TABLE 3:** Ranking of the top 10 co-cited authors with the most citations.

Ranking	Times cited	Author	Affiliation	*h*-Index
1	145	Patrice D. Cani	Universite Catholique Louvain	92
2	119	W. H. Wilson Tang	Cleveland Clinic	81
3	117	Peter J. Turnbaugh	University of California, San Francisco	48
4	104	Ruth E. Ley	Max Planck Institute for Developmental Biology	64
5	98	Zeneng Wang	Cleveland Clinic Foundation	52
6	79	Fredrik Backhed	University of Gothenburg	87
7	70	Anja Sandek	University of Gottingen	24
8	69	Robert A. Koeth	Cleveland Clinic	8
9	67	Junjie Qin	BGI-Shenzhen	12
10	60	J. Gregory Caporaso	Northern Arizona University	63

### Analysis of Co-cited References

**FIGURE 5:**
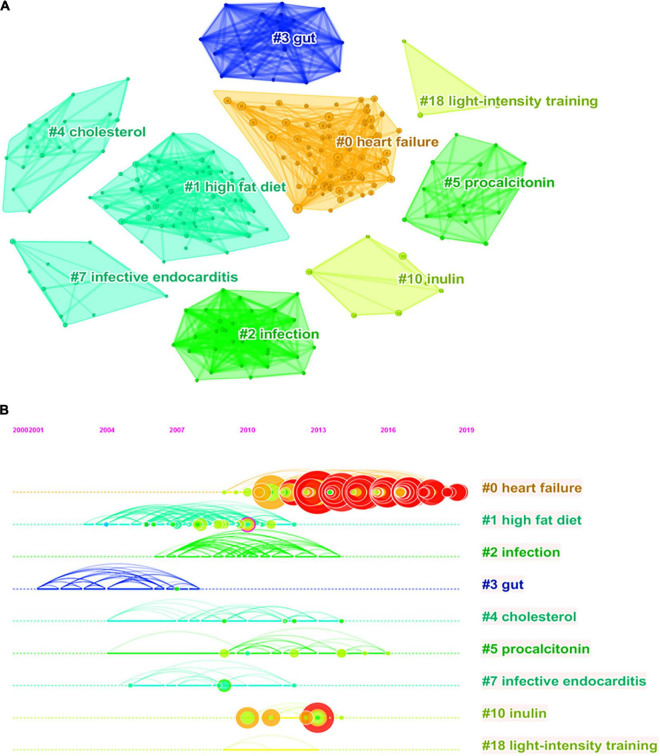
Reference co-citation network knowledge map for research of gut microbiota in the heart failure field from 2006 to 2021. **(A)** Clustering visualization map of the reference co-citation, labeled with the largest nine clusters. **(B)** Timeline visualization map of the reference co-citation.

Analysis of co-cited references reveals the authoritative nature of the research in the field and the great contribution of the authors. Figure 5A shows the clustering visualization map of co-cited references based on the CiteSpace software, which was divided into 114 clusters based on indexing terms, and finally a log-likelihood ratio algorithm (LLR) was used to extract the largest nine clusters. The figure displays them with different convex hulls, including heart failure (cluster #0), high fat diet (cluster #1), infection (cluster #2), gut (cluster #3), cholesterol (cluster #4), procalcitonin (cluster #5), infective endocarditis (cluster #7), inulin (cluster #10), and light-intensity training (cluster #18). Table 4 presents details on the largest nine clusters of references in the co-citation network. In addition, the timeline view is a visualization method that combines the clustering analysis and time-slicing techniques. Figure 5B then shows the largest nine clusters in a timeline view, illustrating the temporal scientific relevance of co-cited references. A total of 398 nodes and 1,512 links was displayed in the visualization map of co-cited references, and the total *Q*-value was 0.8052, harmonic mean (*Q*, *S*) = 0.886, and the mean silhouette value of each cluster was above 0.9, manifesting that the cluster quality was credible and significant.

**TABLE 4:** The largest nine clusters in the reference co-citation network.

Cluster	Size	Mean silhouette	Mean year	Label (LLR algorithm)	Representative Reference
0	72	0.995	2015	Heart failure	Tang et al. (15)
1	60	0.949	2008	High fat diet	Cani et al. (18)
2	32	1	2010	Infection	Sohail et al. (19)
3	19	0.988	2004	Gut	Sandek et al. (20)
4	18	1	2010	Cholesterol	Parnell et al. (21)
5	16	1	2011	Procalcitonin	Schuetz et al. (22)
7	12	1	2008	Infective endocarditis	Habib et al. (23)
10	7	1	2012	Inulin	Everard et al. (24)
18	3	0.987	2011	Light-intensity training	Turnbaugh et al. (25)

*“Size” means the number of references that a cluster contains. “LLR” means log-likelihood ratio.*

As shown in Table 5, the characteristics of the top 10 highly co-cited references concerning gut microbiota research in the heart failure field were summarized. Each of these references was found in cluster #0, with the top-ranked reference being published by Tang et al. (64) (15), followed by Koeth et al. (63) (16), and Tang et al. (58) (17).

**TABLE 5:** Ranking of the top 10 co-cited references for gut microbiota research in the heart failure field.

Ranking	Cited by	References	Title	Source title	Year of publication	Type of document
1	64	Tang et al. (15)	Prognostic value of elevated levels of intestinal microbe-generated metabolite trimethylamine-N-oxide in patients with heart failure: refining the gut hypothesis	Journal of the American College of Cardiology	2014	Article
2	63	Koeth et al. (16)	Intestinal microbiota metabolism of L-carnitine, a nutrient in red meat, promotes atherosclerosis	Nature Medicine	2013	Article
3	58	Tang et al. (17)	Intestinal microbial metabolism of phosphatidylcholine and cardiovascular risk	The New England Journal of Medicine	2013	Article
4	49	Marques et al. (26)	High-fiber diet and acetate supplementation change the gut microbiota and prevent the development of hypertension and heart failure in hypertensive mice	Circulation	2017	Article
5	48	Wang et al. (27)	Gut flora metabolism of phosphatidylcholine promotes cardiovascular disease	Nature	2011	Article
6	47	Yang et al. (28)	Gut dysbiosis is linked to hypertension	Hypertension	2015	Article
7	46	Wang et al. (29)	Non-lethal inhibition of gut microbial trimethylamine production for the treatment of atherosclerosis	Cell	2015	Article
8	45	Pasini et al. (30)	Pathogenic gut flora in patients with chronic heart failure	JACC Heart Failure	2016	Article
9	41	Luedde et al. (31)	Heart failure is associated with depletion of core intestinal microbiota	ESC Heart Failure	2017	Article
10	41	Zhu et al. (32)	Gut microbial metabolite TMAO enhances platelet hyperreactivity and thrombosis risk	Cell	2016	Article

### Analysis of Co-occurring Keywords

**FIGURE 6:**
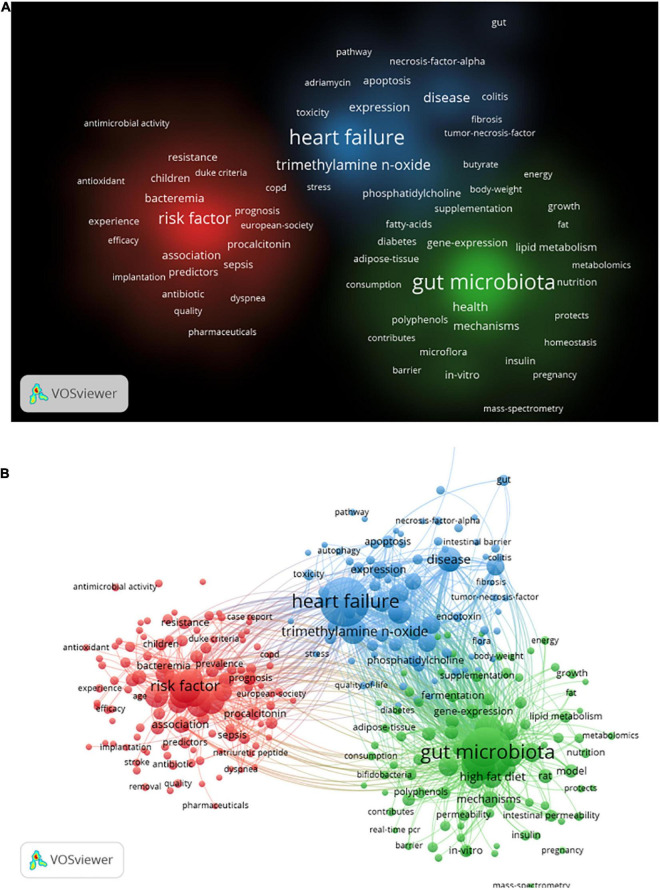
Visualization of keyword co-occurrence analysis from 2006 to 2021 based on the VOS viewer software. **(A)** The density visualization map, and the depth of the color was positively correlated with the occurrences of keywords. **(B)** The network visualization map, all the keywords could be clustered into three categories.

**FIGURE 7:**
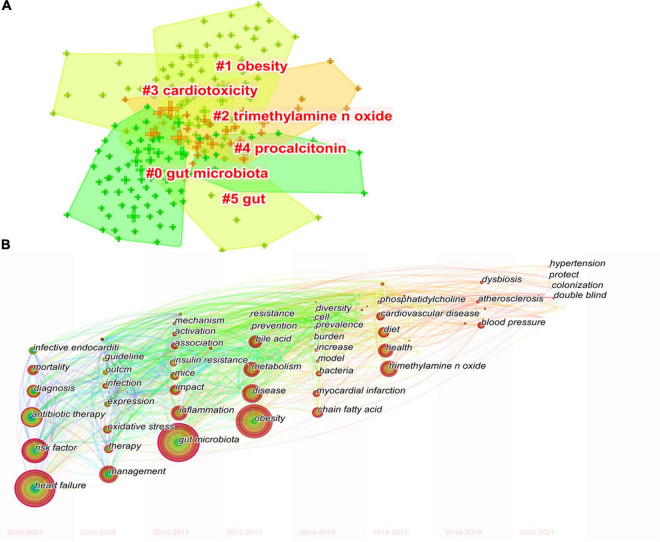
The keywords clustering knowledge map for research of gut microbiota in the heart failure field from 2006 to 2021. **(A)** Clustering visualization map of the co-occurring keywords, labeled with the largest six clusters. **(B)** Time-zone view of co-occurring keywords, with the size of each node proportional to the frequency of keyword occurrences.

Keywords co-occurrence analysis can be used to identify research topics and to analyze research hotspots, as well as to monitor the transition of research frontiers within a certain knowledge domain. VOS viewer was utilized for keyword co-occurrence clusters with a minimum of five occurrences, as shown in Figure 6, the size of each node indicates the occurrence of the keyword. Three clusters were shown in different colors, and nodes with common attributes were classified into a color-coded cluster, represented by green, blue, red, which revolved around gut microbiota, heart failure, and risk factor, respectively. The details of each cluster are shown in Supplementary Figure 6. In addition, a landscape generated using clusters of keywords based on CiteSpace software presents the following six blocks (Figure 7A), the overlapping portions of the image indicate where studies could be classified within more than one cluster. Supplementary Table 2 shows the details of the largest six clusters in co-occurring keywords. In addition, time-zone view of co-occurring keywords are shown in Figure 7B, consisting of 189 nodes and 1,074 links that represent the keywords and their co-occurrence relationships. In terms of co-occurrence frequency, the top 10 ranked keywords are shown in Table 6, including “heart failure,” “gut microbiota,” “obesity,” “risk factor,” “inflammation,” “TMAO,” “metabolism,” “antibiotic therapy,” “management,” and “disease.” Of note are the following, the keywords “Obesity” and “TMAO” have become the top research hotspots since 2012 and 2016 (Figure 7), appearing in, respectively, 122 and 71 citing publications (Table 6).

**TABLE 6:** Ranking of the top 10 keywords for gut microbiota research in the heart failure field in terms of frequency.

Ranking	Keyword	Frequency	Ranking	Keyword	Frequency
1	Gut microbiota	173	6	TMAO	71
2	Heart failure	149	7	Metabolism	70
3	Obesity	122	8	Antibiotic therapy	69
4	Risk factor	93	9	Management	64
5	Inflammation	73	10	Disease	60

### Analysis of Burst Detection

**FIGURE 8:**
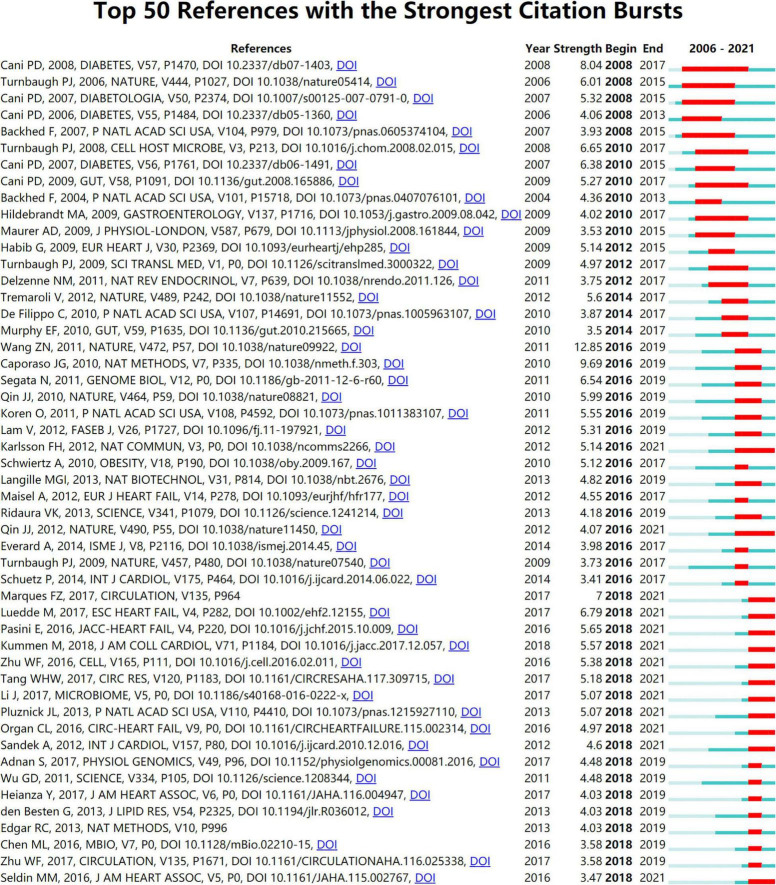
Annual ranking of references with the strongest citation bursts related to gut microbiota research in the heart failure field. The strength values reflect the frequency of citation. *Red* bars indicate a burst period for the references.

Burst detection analysis may identify the emerging concepts and future trends that have caught the attention of peer investigators. The strength of the burst and the duration of the burst state are the two attributes of the citation burst. By using CiteSpace, Figure 8 displays references with the strongest citation bursts during the period of 2006–2021. References with citation bursts first appeared in 2008 (18, 33–36), and the most recent references with citation bursts appeared in 2018 (6, 26, 30–32, 37–49). The strongest burst (strength: 12.85) appeared in 2016 for a 2011 article (27). A total of 13 references had a burst that lasted until 2021 (6, 26, 30–32, 37–41, 49–51). The references with citation bursts between 2010 and 2018 accounted for 90.00%.

**FIGURE 9:**
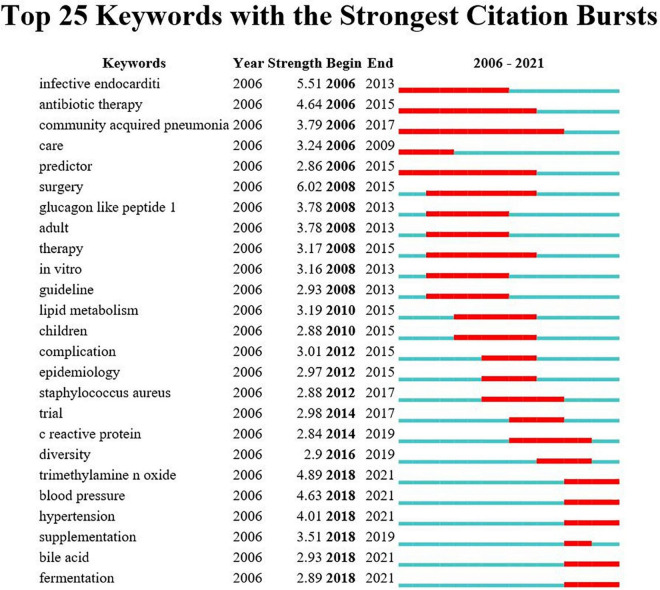
Annual ranking of keywords with the strongest citation bursts related to gut microbiota research in the heart failure field. The strength values reflect the frequency of citation. *Red* bars indicate a burst period for the keywords.

Figure 9 shows the top 25 keywords with citation bursts. Their stronger burst rate indicates greater attention to the research topic, which can be a good indicator of the research frontier in this period. Over the past decade, surgery ranked first with the highest burst strength (strength: 6.02), followed by infective endocarditis (strength: 5.51), TMAO (strength: 4.89), antibiotic therapy (strength: strength: 4.64), and blood pressure (strength: 4.63). More importantly, TMAO, blood pressure, hypertension, supplementation, bile acid, and fermentation became the focus from 2018 until now, indicting they were current research hotspots.

## Discussion

With the rapid increase in the number of deaths from heart failure each year, the search for possible factors and new therapeutic strategies has become imperative. It has been reported that an imbalance between the gut microbiota alter the microecological environment of a gastrointestinal tract, resulting in numerous diseases. Since then, gut microbiota has attracted the attention of researchers. Is modulating gut microbiota to restore injured heart function a possible therapeutic strategy? Over the past nearly two decades, researchers have made extensive efforts to elucidate the link between them. With the help of scientometric investigation, it is possible to provide the history and current status of research in the subject area, and to predict future research directions. To best of our knowledge, this is the first-ever study to provide systematic information on the link between gut microbiota and heart failure through bibliometric analysis coupled with visualized mapping. Researcher may benefit from these results by gaining a basic understanding and identifying areas of interest or trends, encouraging them to conduct further research in this field.

### General Information

On the basis of the WoSCC database, we analyzed a total of 873 literature studies that were indexed by SCIE from 2006 to 2021, of which 81.79% were original articles and 18.21% were reviews. As evidenced by a qualitative and quantitative analysis conducted using online platform, VOS viewer and CiteSpace software, the number of scientific research outputs pertaining to the link between gut microbiota and heart failure has increased significantly during the past 16 years. The United States, which lead in the number of publications, also had a maximum centrality of 0.45, and was the country that plays a central role in promoting cooperation among individual countries. Remarkably, China, as a developing country, had shown tremendous progress in this field with 167 relevant articles, Zhu et al. (32, 48) and Qin et al. (50) had published a series of articles around the link between microbial products (e.g., TMAO) and heart failure. As for the authorship, Nathalie M. Delzenne, Audrey M. Neyrinck, and Patrice D. Cani had established a relatively stable collaboration, ranking first, second, and fourth in terms of number of publications, respectively. However, we realized that the collaborative efforts among top researchers seem to be insufficient, and that countries and institutions should encourage scholars to actively increase their collaborations and publish higher quality articles.

Analysis journal and co-cited journals can provide important information for researchers to choose the proper journal in which to submit their manuscripts. Most relevant studies were published in Q1 journals, while those with more co-citations appeared in the journals with world-class influence, such as *New England Journal of Medicine*, *Nature*, *Circulation*, *Lancet*, and *Science*. These results suggested that the link between heart failure and gut microbiota has attracted the attention of numerous scholars, and its research difficulties and value have also been recognized by scholars. However, only a 10% concordance rate was observed between the top 10 most active journals and the top 10 co-cited journals, indicating a need to improve the quality of research in this field, as well as to strengthened international collaboration among scholars to produce high-quality research.

The published literature studies focused on cardiac cardiovascular systems, microbiology, and pharmacology pharmacy, as well as infectious diseases, biochemistry molecular biology, food science technology, nutrition, reflecting the multidisciplinary intersection that is a characteristic of research in this field. Multidisciplinary intersection will aid in breaking through the technical condition limitation of the research between gut microbiota and heart failure, and will provide the impetus for the development of this field.

### Knowledge Base

Analysis of reference co-citation networks showed that all the top 10 highly co-cited references fell under the largest theme cluster #0 (“heart failure”). Among the top 10 co-cited references, the first and third were both published by Professor W. H. Wilson Tang group in *JACC* 2013 and *The New England Journal of Medicine* 2014 (15, 17), respectively, where the authors further found that patients with heart failure had elevated TMAO levels compared to patients without heart failure, and elevated TMAO levels were associated with higher long-term mortality risk in spite of traditional risk factors and cardiorenal indexes, with landmark implications. The second most co-cited reference was published by Robert A. Koeth in *Nature Medicine* in 2013 (16), in which it was demonstrated that gut microbiota metabolism of L-carnitine plays an important role in atherosclerosis pathogenesis and suggested new potential therapeutic targets for preventing cardiovascular disease. Moreover, cluster #1 was a collection of studies on high-fat diets, a representative study showing that high-fat feeding alters intestinal flora, which in turn increases intestinal LPS permeability, and several inflammatory markers were analyzed and mRNA expressions of PAI-1, TNF-α, and IL-1 were found to be completely eliminated by antibiotic treatment after high-fat feeding (18). Furthermore, high-fat diets lead to abnormal glucose metabolism and cardiac tissue damage through uric acid-dependent mechanisms, whereas butyric acid (a type of SCFAs) protects against high fat diet-induced cardiometabolic disturbances by inhibiting uric acid and enhancing glutathione antioxidant defenses (52). Cluster #10 focused on elucidating the role of inulin in the gut microbiota, and studies have shown that inulin-type fructans (ITFs) have been implicated as regulators of microbial ecology and host physiology in animals and humans. Rodent models of genetic and diet-induced obesity also showed ITF to be beneficial to reducing body weight gain and fat mass accumulation, improving glucose tolerance and insulin resistance, improving intestinal barrier function, and reducing inflammation (53). In addition, other highly co-cited references shed light on the possible link between heart failure and gut microbiota around high-fat diet (26), phosphatidylcholine (17, 27), cholesterol, and high-intensity training.

As shown in keywords clustering map in Figure 6, it was observed that all the keywords could separate into three clusters, besides the subject terms “heart failure” and “gut microbiota,” it is more important to focus on the role of “risk factor,” for example, studies have shown comorbidities such as diabetes or chronic kidney disease are a high risk for heart failure patients, and that antibiotic therapy may be a potential treatment by modification of the gut microbiota (Supplementary Figure 6). More interestingly, as shown by the clustering visualization and time-zone view of co-occurrence keywords (Figure 7), early studies of gut microbiota in patients with heart failure focused on removing gut decontamination with broad-spectrum antibiotics in an attempt to reduce inflammation levels and bacterial translocation. Infective endocarditis is characterized by a high rate of staphylococcus aureus infection, which leads to a relatively high rate of development of moderate-to-severe heart failure (54, 55). Similarly, Sandek et al. also observed bacterial overgrowth in patients with heart failure that consisted of mucosal biofilm with increased bacterial adhesion, which could further lead to chronic inflammation and malnutrition (20, 56). Probiotic supplement studies reported that administration of *Lactobacillus plantarum* 299v decreased circulating leptin levels, reduced myocardial infarcts and improved ventricular function and remodeling after left anterior descending artery ligation (57), and another study showed that saccharomyces boulardii was effective in improving left ventricular ejection fraction and left atrial diameter in patients with chronic heart failure (58). With the in-depth study of gut microbiota, metabolites represented by TMAO and SCFA have played an important role in promoting the progression of heart failure and other cardiovascular disease. In 2017, Marques et al. demonstrated the importance of consuming a high-fiber diet and supplementing with SCFAs to lower blood pressure, minimize cardiac remodeling, and inhibit cardiac hypertrophy and fibrosis through the modification of gut microbial modulation (26). Evidence from a meta-analysis by Zheng et al. suggested that high long-chain omega-3 polyunsaturated fatty acids (LC n-3 PUFAs), obtained in the diet from seafood or in the form of supplements, may have preventive effects on heart failure (59). Therefore, the modulation of dietary composition and intestinal metabolites are expected to be new therapeutic targets of heart failure treatment.

### Emerging Topics

The dynamic nature of trends in this field are partially characteristic of the references with citation bursts. Statistics from CiteSpace found that the first representative reference with the strongest currently ongoing citation bursts was an article published by Wang et al., which illustrated that elevated plasma levels of the three dietary lipid phosphatidylcholine metabolites (namely choline, TMAO, and betaine) were associated with increased cardiovascular risk based on metabolomics studies (27). The second highest burst of reference focused on big data mining software in microbiology, introducing quantitative insights into microbial ecology (QIIME), a microecological sequencing data analysis process developed by Caporaso et al. and published in the *Nature Methods* in 2010 (60), which laid the methodological foundation for the link between gut microbiota and heart failure. The software has become the most widely used analysis tool in the microbiota field, with over 10,000 citations, and was named one of the 25 milestone events in human microbiota research in the last 70 years by *Nature*. Furthermore, the publication with the third strongest citation bursts, by Cani et al. (18), was published in 2008, in the *Diabetes* with the burstness strength of 8.04, and the burstness has lasted for 10 years (2008–2017). This new finding implies that intestinal permeability may be increased by changes in gut microbiota, which in turn may control metabolic endotoxemia, inflammation, and associated disorders (18). The citation burstness analysis showed that exploring the link between gut microbiota and heart failure is multi-disciplinary and needs to be explored with the help of emerging research tools, and it is believed that the future development of multi-omics association and artificial intelligence technologies may breathe new life into this field.

These research hotspots have revealed many novel findings that have contributed to a rise in publications. The “burst keywords” can be categorized into three phases based on when they started and when they ended. The first stage included “infective endocarditis,” “antibiotic therapy,” “community acquired pneumonia,” “care,” and “predictor,” these disease extensions suggest that gut microbiota may be associated with heart failure. The second stage included “glucagon like peptide 1,” “lipid metabolism,” “staphylococcus aureus,” “c reactive protein,” etc., elucidating the possible influence of inflammatory indicators and various disorders of glycolipid metabolism on the pathogenesis of heart failure, and paving the way for the study of specific mechanisms of gut microbiota in heart failure. The third stage, that is, the keywords with high burst intensity that started in 2018 and have currently ongoing bursts, specifically contain “TMAO,” “blood pressure,” “hypertension,” “bile acid,” and “fermentation.” A growing body of literature suggest that modulation of intestinal metabolites (TMAO, bile acid, and SCFAs) holds promise as new therapeutic targets for heart failure patients, which was consistent with the strongest citation keywords burst of this study in 2018. Animal experiments in heart failure from Organ et al. found that either choline supplementation, or direct TMAO feeding, could result in higher levels of systemic TMAO, aggravated myocardial fibrosis, as well as worsened hemodynamic and anatomic parameters in mice after trans-aortic constriction (TAC) (41). Beyond animal model studies showing heightened TMAO is associated with worse adverse ventricular remodeling and function in heart failure models, clinical data also revealed that higher TMAO levels were associated with poorer prognoses with respect to all-cause mortality, hospitalization, and heart transplantation for patients with heart failure (61). And even one study showed that TMAO was a better predictor of prognosis than BNP (62). Coincidentally, Suzuki et al. in patients with heart failure showed that the inclusion of TMAO in the risk model, combined with clinical scores, improved risk stratification for in-hospital mortality, while the combination of TMAO and NT-proBNP would provide additional prognostic predictive information (63). Meanwhile, clinical trial studies have found that the application of antibiotic intervention reduces TMAO synthesis and thus myocardial hypertrophy and myocardial fibrosis (64). Further extension of the findings of the relationship between TMAO levels and cardiovascular disease, some studies had also demonstrated that plasma TMAO levels were associated with the risks of incident thrombotic event and had a degree of elevating effect on blood pressure (32, 48). Bile acids are another important metabolite of gut microbiota, a cross-sectional study showed an increased ratio of secondary to primary bile acids in the serum of chronic heart failure patients, and univariate analysis showed that this ratio was associated with a decrease in overall survival (3). It has been shown that bile acid responsive receptors, namely TGR5 agonists or FXR inhibitors, exert cytoprotective effects on the heart by improve myocardial responses to physiological, inotropic, and hemodynamic stress (65, 66). Meanwhile, an increasing number of emerging molecules have been shown to be independent novel biomarker of heart failure risk stratification, such as secreted frizzled-related protein 2 (SFRP2) and SFRP5 (2, 67), and it is believed that future application with intestinal metabolites will be able to better predict the development of heart failure.

Research conducted in recent years had found that metabolic disorders can have unhealthy effects on cardiovascular health, which include hypertension, atherosclerosis, heart failure, dyslipidemia, diabetes, and chronic kidney disease as well (38). A high prevalence of hypertension is particularly prevalent in heart failure patients, as 91% had underlying hypertension at the time of their diagnosis (68). Therefore, it is necessary to study how gut microbiota affects the regulation of blood pressure. As a result of experimental hypertension, spontaneous hypertension rats were found to have significantly higher plasma levels of TMA than normotensive rats, and enalapril as a hypotensive agent was able to significantly reduce the plasma levels of TMA as well (69). Other studies had demonstrated that SCFAs were correlated with blood pressure levels in the pathogenesis of hypertension, and SCFAs may lower blood pressure by regulating vasodilation, specifically possibly through the G protein-coupled receptor orphan type (Gpr41) and the olfactory receptor 78 (Olfr78), which exert blood pressure modulating effects. Similar studies related to co-morbidities such as diabetes and chronic kidney disease are increasingly being conducted, and although there is the large body of evidence to date pointing to TMAO and SCFAs as the major gut metabolites involved in heart failure and co-morbidities such as diabetes and chronic kidney disease (63, 70–72), other dietary nutrients and fermentation metabolites should not be ignored (73).

Currently, the supplementation or improvement of gut microbiota has become a frontier topic in the field of heart failure, including dietary interventions, antibiotic interventions, probiotic and prebiotic therapy, faecal microbiota transplantation (FMT), TMA-lyase inhibitors, etc. (74). It is possible to improve the intestinal flora structure of heart failure by transplanting beneficial bacteria, low yielding TMAO intestinal flora, and high yielding SCFAs intestinal flora. However, whether FMT can improve heart failure by restoring the diversity and function of the intestinal flora still needs extensive experimental and clinical studies.

Hence, in the future, to better understand this multi-layered complex gut ecological dysregulation-heart failure relationship, it will be necessary to study larger sample sizes and broader populations, as well as to adjust for various factors and medications that may affect the growth of gut bacterial. Further, to make sense of the vast amount of data generated by these studies, artificial intelligence techniques such as deep learning neural networks or others may be needed to help decipher meaningful patterns.

### Strengths and Limitations

To our knowledge, this scientometric investigation is the first of its kind to identify and characterize the association with gut microbiota and heart failure. The application of scientometric provides clearer insights into the evolving focuses and trends in research than traditional narrative reviews. Researchers could utilize these information to identify new research directions and explore potential cooperation opportunities in this field. However, there are several limitations to our study. Firstly, the data collection was only retrieved from the WoSCC database, potentially resulting in bias and incompleteness in the included studies. Additionally, for the purposes of better presentation of the results and guaranteeing the quality of all included literature studies, we only included articles and reviews published in English. Finally, despite our normalization procedures, bias may have still existed because some authors have the same name, and some keywords are expressed differently, the database is constantly updated, etc. These limitations may be addressed in a better way in similar studies in the future. Despite these limitations, the findings are still considered to be an effective representation of the research output of gut microbiota research in the heart failure field on a global scale.

## Conclusion

This study shows a gradual expansion of worldwide research on gut microbiota and heart failure from 2006 to 2021, indicating a well-developed and promising research field. The most frequent subject category was cardiac cardiovascular systems. The United States constituted the core research forces. Strikingly, the cooperation between the institutes and authors were relatively weak. Further advances in this field will make significant breakthroughs in the development of novel therapeutic tools for metabolic modulation of heart failure. In conclusion, this is the first scientometric analysis that provides a comprehensive overview of research on the link between gut microbiota and heart failure, which could help us realize the main research institutions and authors, core journals, evolutionary pathways, frontier research hotspots, and future trends in this field.

## Data Availability Statement

The original contributions presented in the study are included in the article/Supplementary Material, further inquiries can be directed to the corresponding authors.

## Author Contributions

FM, JW, and HT conceived and designed the study. FM wrote the original draft preparation. YG and RL assisted in literature searching. MT conducted an analysis based on WoS. MT, HZ, and FM conducted the software analysis. MZ, JZ, and SH provided the figures and tables. FM, MT, YG, and RL reviewed and edited the manuscript for content and style. All authors contributed to the article and approved the submitted version.

## Conflict of Interest

The authors declare that the research was conducted in the absence of any commercial or financial relationships that could be construed as a potential conflict of interest.

## Publisher’s Note

All claims expressed in this article are solely those of the authors and do not necessarily represent those of their affiliated organizations, or those of the publisher, the editors and the reviewers. Any product that may be evaluated in this article, or claim that may be made by its manufacturer, is not guaranteed or endorsed by the publisher.

## Footnotes

1
http://bibliometric.com/

## Supplementary Material

Supplementary Figure 1 Conceptual design of the study.

Supplementary Figure 2 The cross-country collaborations visualization map was generated by the online bibliometric analysis.

Supplementary Figure 3 Visualization knowledge maps of the scientific collaboration network based on the VOS viewer software. **(A)** Inter-country collaboration network map. **(B)** Inter-institutional collaborative network map. **(C)** Inter-author collaborative network map.

Supplementary Figure 4 Visualization collaboration network of co-citation journal from 2006 to 2021 based on the CiteSpace software.

Supplementary Figure 5 Visualization collaboration network of co-citation author from 2006 to 2021 based on the CiteSpace software.

Supplementary Figure 6 Specific details of the network visualization map of keyword co-occurrence analysis based on the VOS viewer software.

Supplementary Table 1 Search strategy in Web of Science Core Collection (January 14, 2022).

Supplementary Table 2 The details of the largest six clusters in co-occurring keywords.
