# Supplementary figures and images for: Knowledge Mapping of the Links Between the Gut Microbiota and Heart Failure: A Scientometric Investigation (2006–2021)
Source: Front Cardiovasc Med. 2022 Apr 28;9:882660. doi: 10.3389/fcvm.2022.882660 (PMC9095927; doi:10.3389/fcvm.2022.882660)

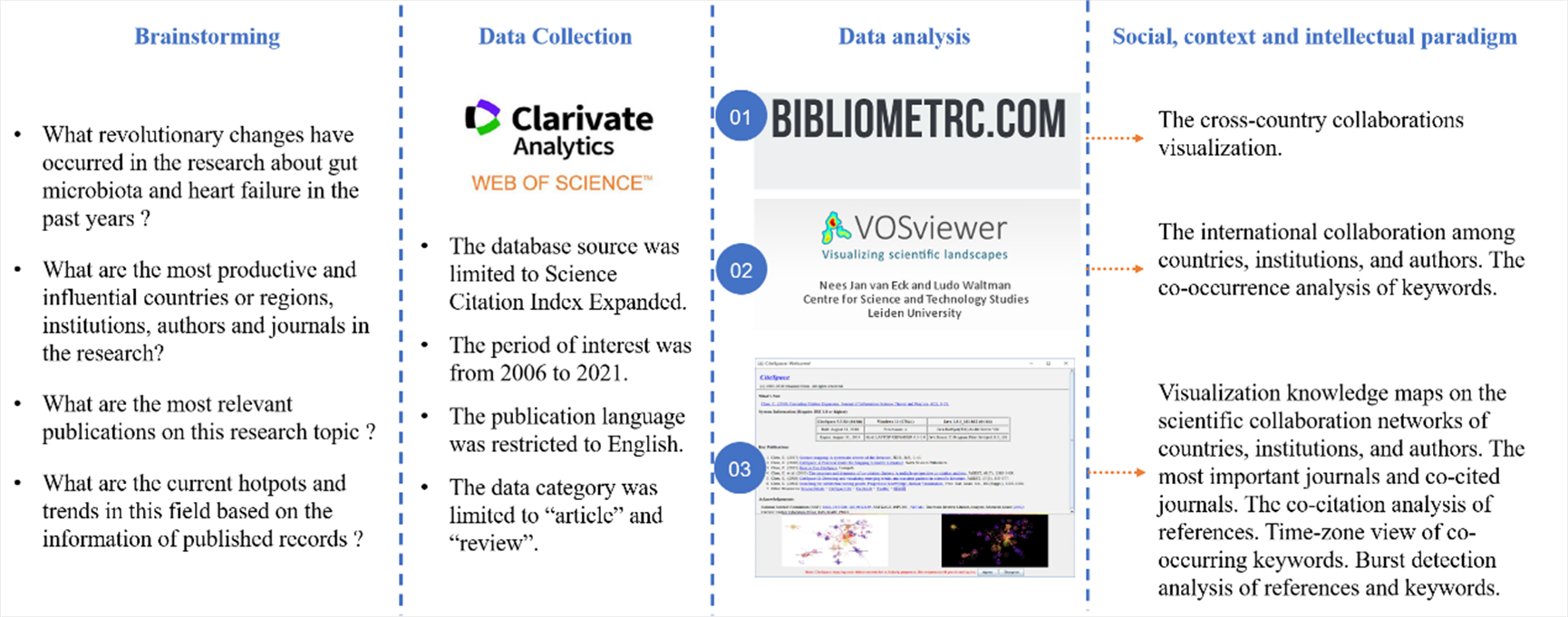

Supplement: Supplementary Figure 1 — Conceptual design of the study. [file Image_1.tif]

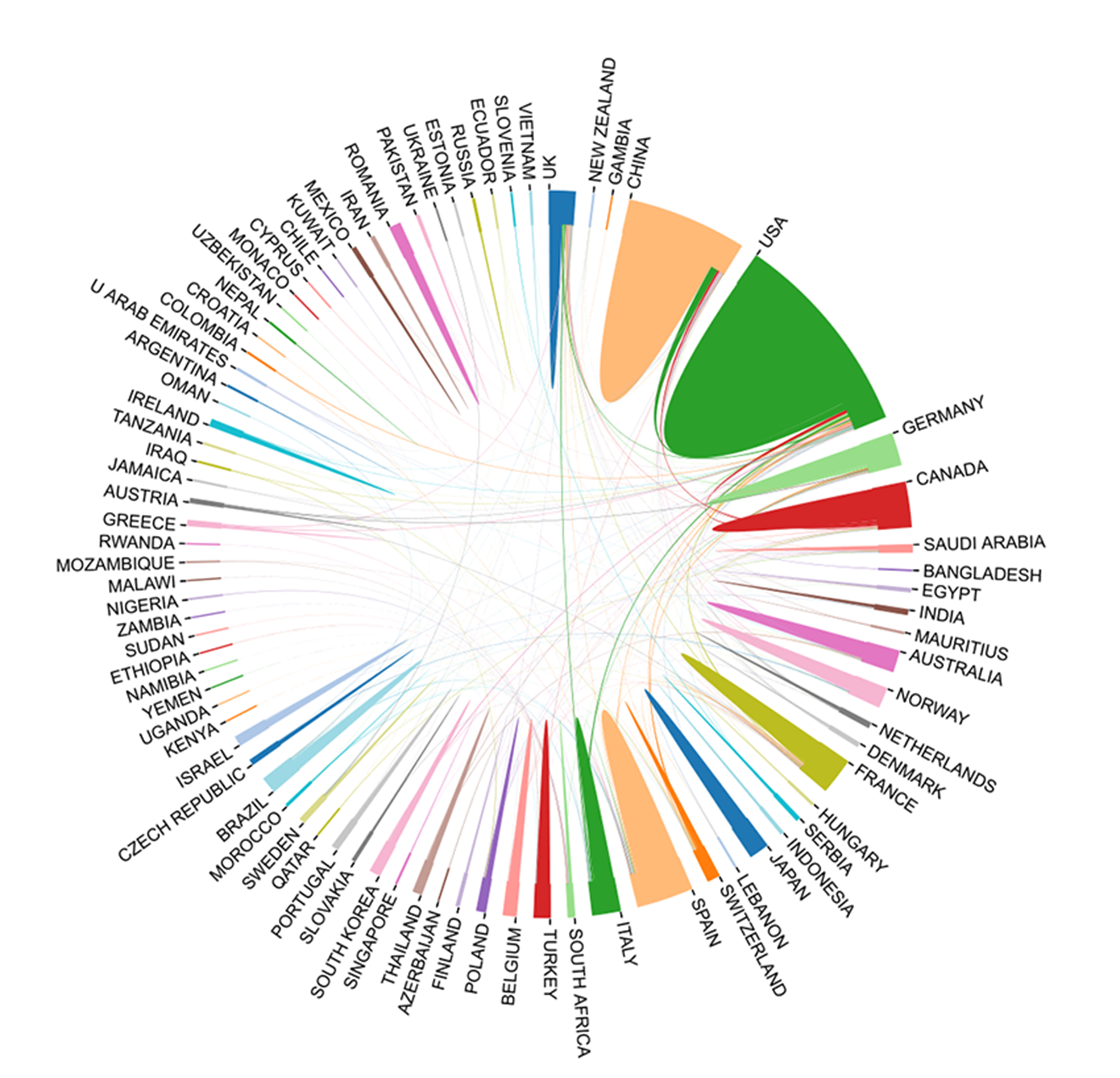

Supplement: Supplementary Figure 2 — The cross-country collaborations visualization map was generated by the online bibliometric analysis. [file Image_2.tif]

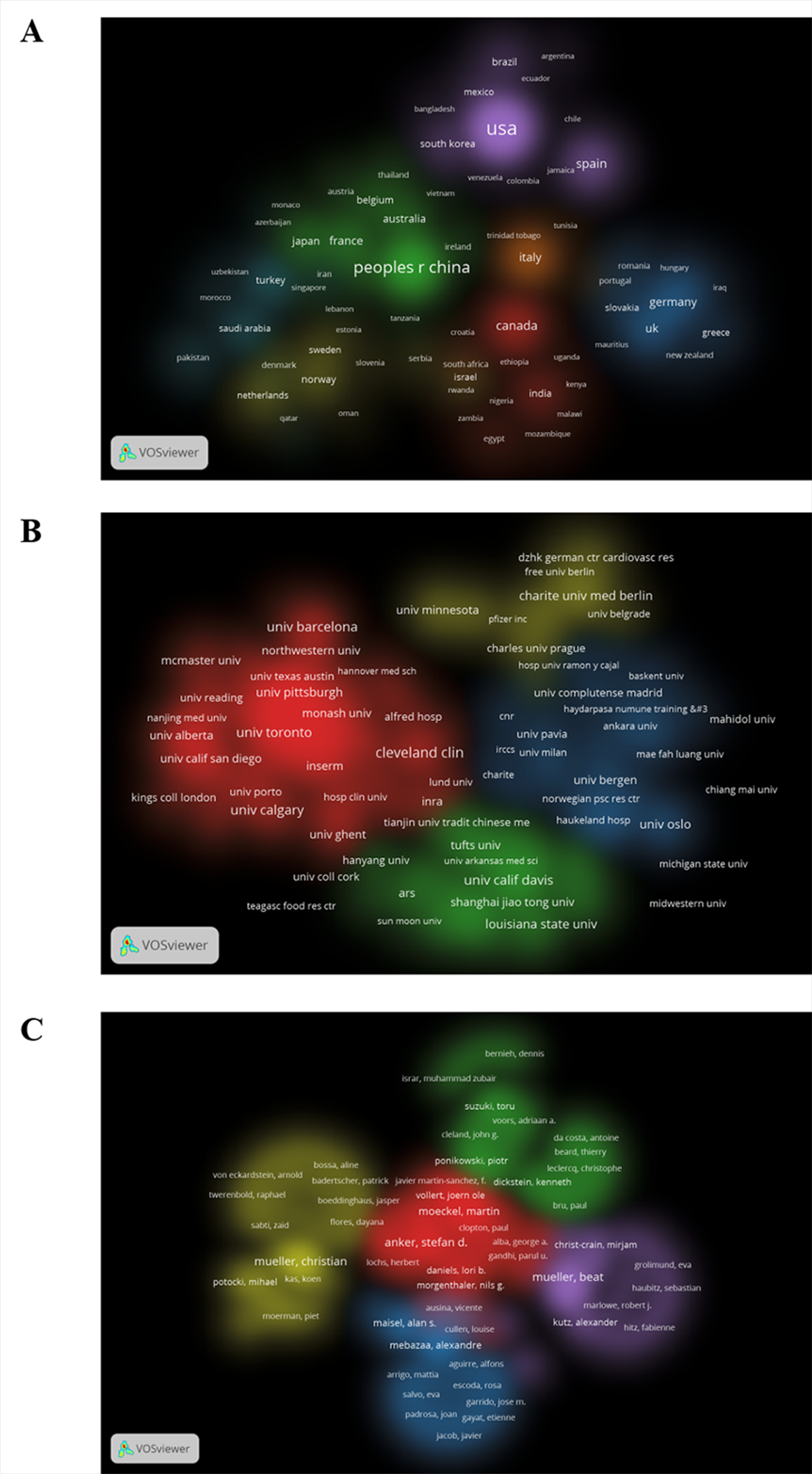

Supplement: Supplementary Figure 3 — Visualization knowledge maps of the scientific collaboration network based on the VOS viewer software. (A) Inter-country collaboration network map. (B) Inter-institutional collaborative network map. (C) Inter-author collaborative network map. [file Image_3.tif]

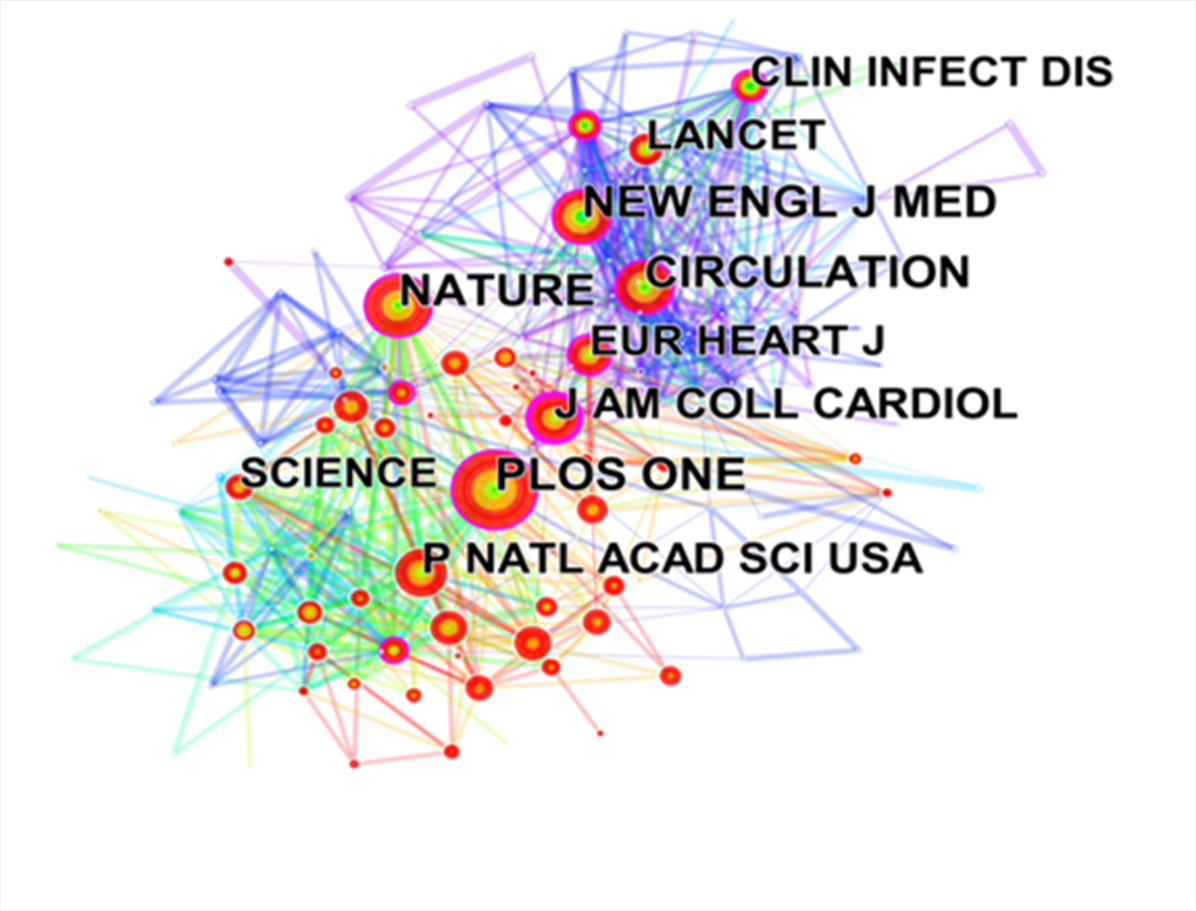

Supplement: Supplementary Figure 4 — Visualization collaboration network of co-citation journal from 2006 to 2021 based on the CiteSpace software. [file Image_4.tif]

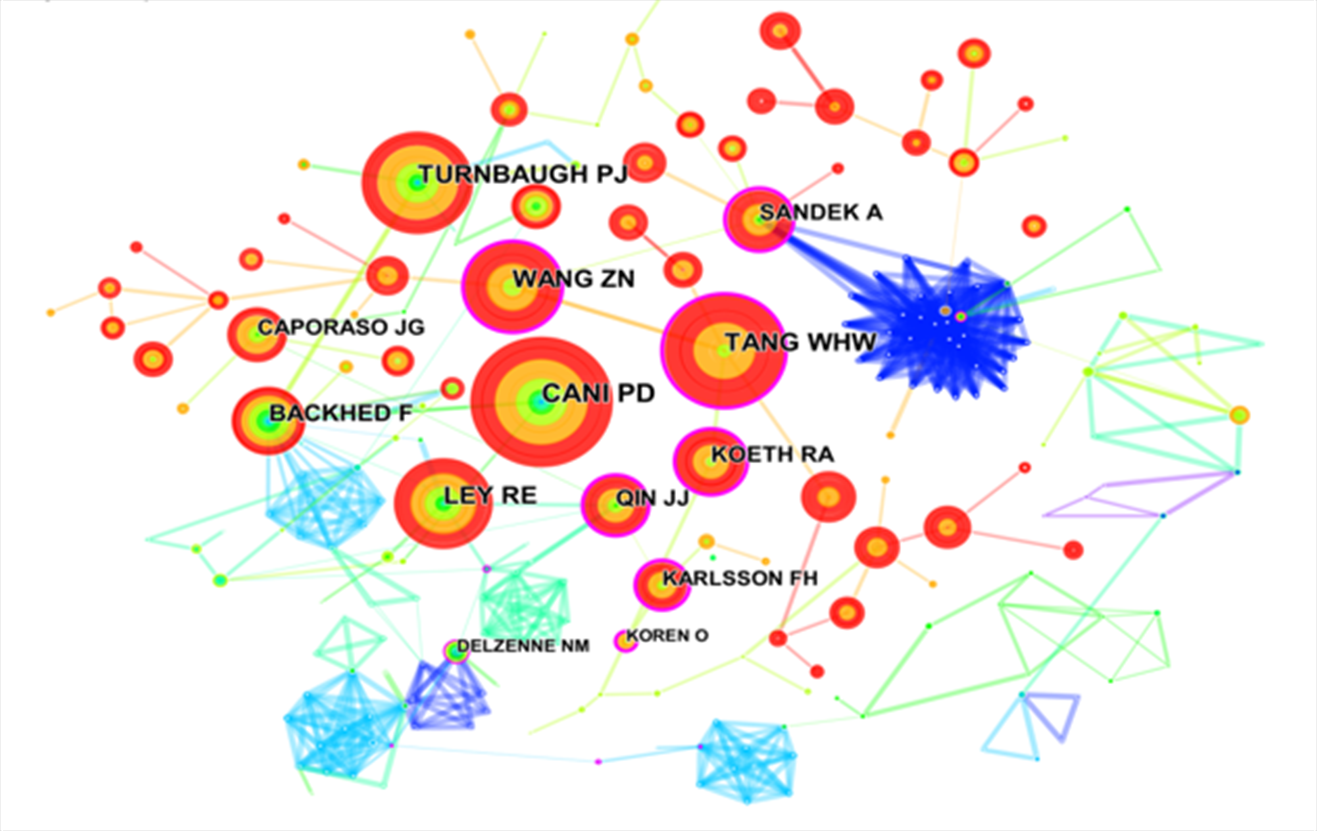

Supplement: Supplementary Figure 5 — Visualization collaboration network of co-citation author from 2006 to 2021 based on the CiteSpace software. [file Image_5.tif]

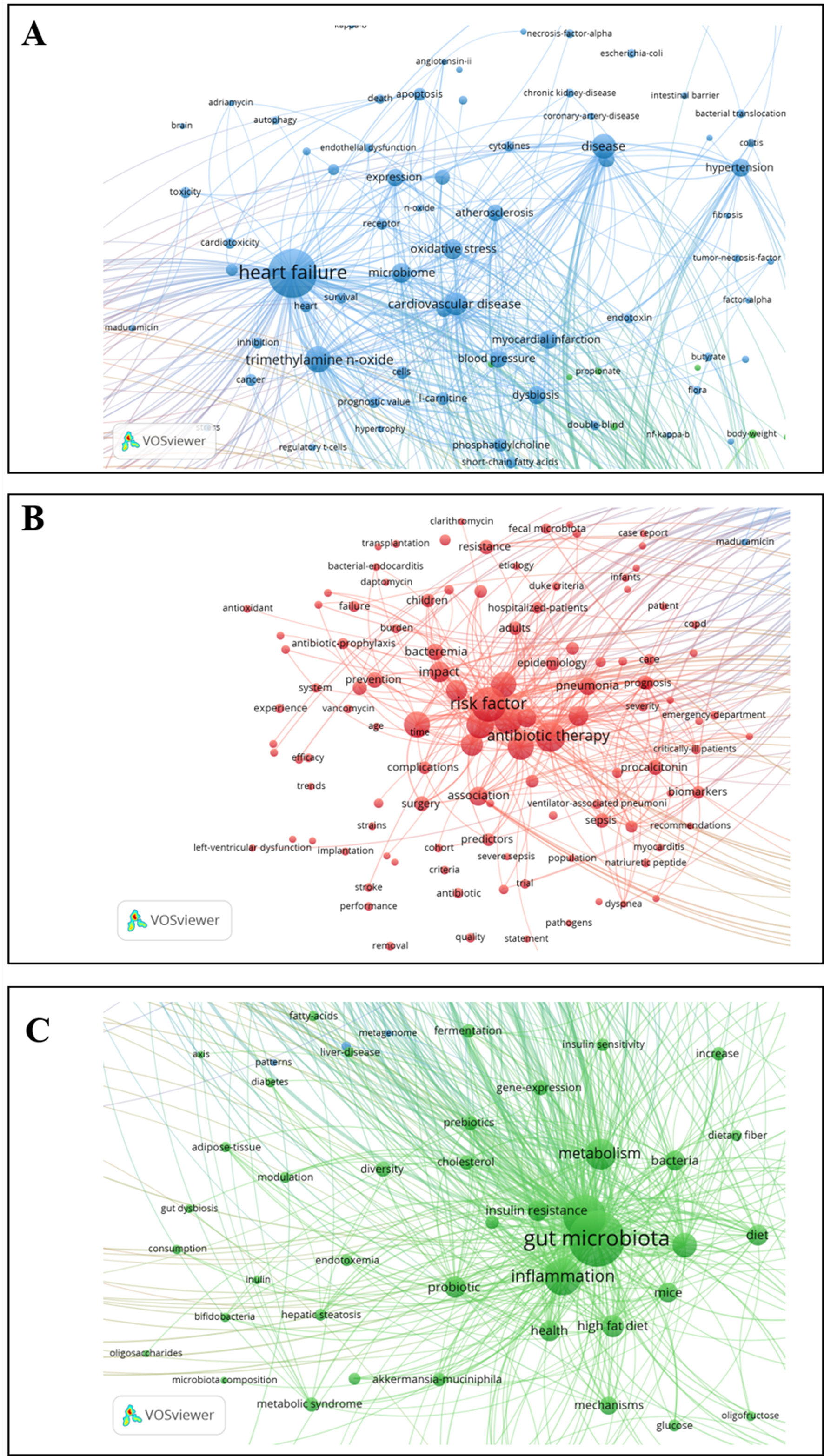

Supplement: Supplementary Figure 6 — Specific details of the network visualization map of keyword co-occurrence analysis based on the VOS viewer software. [file Image_6.tif]
