# Supplementary material for: Knowledge Mapping of the Links Between the Gut Microbiota and Heart Failure: A Scientometric Investigation (2006–2021)
Source: Front Cardiovasc Med. 2022 Apr 28;9:882660. doi: 10.3389/fcvm.2022.882660 (PMC9095927; doi:10.3389/fcvm.2022.882660)
Supplement: Supplementary Table 1 — Search strategy in Web of Science Core Collection (January 14, 2022). [file Table_1.docx]

**TABLE S1 |** Search strategy in Web of Science Core Collection (Jan 14, 2022).

| TS = (((“gut” OR “gastrointestin∗” OR “intestin∗” OR “gastro-intestin∗”) AND (“microbiot∗” OR “flora” OR “bacteria” OR “microflora” OR “microbiome∗”)) OR “dysbiosis” OR “probiotic” OR “prebiotic” OR “antibiotic”) AND TS=(“heart failure∗” OR “myocardial failure∗” OR “cardiac failure∗” OR “heart decompensation∗” OR “HF∗”) |
| --- |
| Note: Wildcard “∗” indicates any group of characters or no character in a pattern (for instance, “microbiot∗”would also return “microbiota” or “microbiotic”). |
