# Supplementary material for: Knowledge Mapping of the Links Between the Gut Microbiota and Heart Failure: A Scientometric Investigation (2006–2021)
Source: Front Cardiovasc Med. 2022 Apr 28;9:882660. doi: 10.3389/fcvm.2022.882660 (PMC9095927; doi:10.3389/fcvm.2022.882660)
Supplement: Supplementary Table 2 — The details of the largest six clusters in co-occurring keywords. [file Table_2.docx]

**TABLE S2 |** The details of the largest six clusters in co-occurring keywords.

| **Cluster** | **Size** | **Mean silhouette** | **Mean year** | **Label (LLR algorithm)** |
| --- | --- | --- | --- | --- |
| 0 | 60 | 0.788 | 2011 | Gut microbiota |
| 1 | 51 | 0.666 | 2013 | Obesity |
| 2 | 31 | 0.798 | 2015 | TMAO |
| 3 | 20 | 0.709 | 2012 | Cardiotoxicity |
| 4 | 13 | 0.847 | 2014 | Procalcitonin |
| 5 | 10 | 0.852 | 2013 | Gut |
